# Comparative characterization of oral and cloacal microbiomes in captive adult and juvenile coconut lorikeets (*Trichoglossus haematodus*) using full-length 16S rRNA sequencing

**DOI:** 10.14202/vetworld.2026.2117-2132

**Published:** 2026-05-18

**Authors:** Rini Rachmatika, Siti Nuramaliati Prijono, Ki Ageng Sarwono, Suhendra Pakpahan, Andri Permata Sari, Sinta Maharani, Sugiyono Saputra, Ainissya Fitri, Roni Ridwan, Wahju Widodo, R Taufiq Purna Nugraha, Windri Handayani, Luthfiralda Sjahfirdi

**Affiliations:** 1 Research Center for Applied Zoology, Research Organization for Life Sciences and Environment, National Research and Innovation Agency (BRIN), Bogor, Indonesia; 2 Research Center for Applied Microbiology, Research Organization for Life Sciences and Environment, National Research and Innovation Agency (BRIN), Bogor, Indonesia; 3 Research Center for Biota System, Research Organization for Life Sciences and Environment, National Re-search and Innovation Agency (BRIN), Bogor, Indonesia; 4 Department of Biology, Faculty of Mathematics and Natural Sciences, Universitas Indonesia, Depok, Indonesia

**Keywords:** age-related variation, avian microbiome, captive breeding, cloacal microbiota, coconut lorikeet, microbial diversity, nectarivore, oral microbiota

## Abstract

**Background and Aim:**

The coconut lorikeet (*Trichoglossus haematodus*) is a nectarivorous parrot species of conservation concern in Indonesia, where captive breeding programs are increasingly implemented to reduce pressure on wild populations. Dietary modifications in captivity may influence host-associated microbiota, which play a critical role in health, nutrition, and adaptation. This study aimed to characterize and compare the oral and cloacal microbiomes of adult and juvenile *T. haematodus* using full-length 16S rRNA sequencing to elucidate age- and site-specific microbial patterns.

**Materials and Methods:**

Six clinically healthy captive *T. haematodus* (three adults and three juveniles) were maintained under standardized environmental and dietary conditions. Oral and cloacal swabs were collected, yielding twelve samples, which were subsequently pooled into four groups: adult oral (AO), adult cloaca (AC), juvenile oral (JO), and juvenile cloaca (JC). DNA was extracted and subjected to full-length 16S rRNA sequencing using Oxford Nanopore Technology. Bioinformatic analyses included taxonomic classification, alpha diversity (Observed operational taxonomic unit (OTU), abundance-based coverage estimator (ACE), Simpson, Fisher)), and beta diversity (Venn diagram and principal coordinates analysis).

**Results:**

A total of 1859 bacterial species were identified across all groups. Microbial composition differed markedly by age and anatomical site. Cloacal samples in both adults and juveniles were dominated by *Rosenbergiella*, with higher abundance in adults (~42%) than juveniles (~24%). Oral microbiota showed greater diversity, with *Alcaligenes* predominating in adults and *Psittacicella* in juveniles. Alpha diversity indices indicated higher richness in juvenile cloacal and AO samples, whereas adult cloacal samples exhibited lower diversity. Beta diversity analysis demonstrated clear separation among groups, indicating distinct microbial community structures influenced by both age and sampling site. Core microbiota shared across groups were limited, with substantial unique operational taxonomic units in each category.

**Conclusion:**

This study provides the first comprehensive characterization of oral and cloacal microbiomes in captive *T. haematodus*. Microbial diversity and composition are strongly influenced by age and anatomical location, with cloacal microbiota showing greater stability and oral microbiota reflecting dietary and developmental differences. The dominance of nectar-associated bacteria such as *Rosenbergiella* highlights the ecological linkage between host diet and microbiome. These findings offer valuable insights for optimizing captive nutrition, improving health monitoring, and supporting conservation strategies for nectarivorous parrots.

## INTRODUCTION

Coconut lorikeets (*Trichoglossus haematodus*) are among the bird species commonly traded both in Indonesia and internationally. Therefore, structured breeding efforts are essential to reduce exploitation pressure on wild populations. In captive breeding systems, providing substitute feed that closely resembles the natural diet is critical, particularly through alternative nectar and protein sources. For example, the diet of captive-bred coconut lorikeets often contains higher protein levels to meet nutritional demands, even though their natural diet is relatively low in protein [1, 2]. This difference highlights the adaptive capacity of *T. haematodus* to acclimatize to varying environmental conditions. When nectar availability is limited, these birds can supplement their diet with alternative food sources [3].

Such dietary modifications can directly influence host-associated microorganisms. Previous studies have reported only minor differences in gut microbiota between wild and captive birds [4]. However, microbiome composition and diversity are strongly influenced by anatomical site, diet, and age [5, 6]. Adaptations in the digestive system of lorikeets include a less muscular gizzard and a shorter intestine compared with granivorous and frugivorous parrots [7]. Their ability to digest sucrose is determined by gut sucrase activity [8, 9]. Under high sugar concentrations, rainbow lorikeets preferentially consume hexose over sucrose to optimize digestion [9, 10]. This dietary selectivity may influence foraging behavior, as birds select flowers with optimal sugar composition. Consequently, the abundance and distribution of flowering plants play a crucial role in survival and reproduction, thereby influencing feeding patterns [3].

Microorganisms contribute significantly to host physiology by enhancing growth, stress tolerance, and feed efficiency [11, 12]. In many species, the oral microbiome serves as an indicator of oral and systemic health [13–15], while the gut microbiome is closely linked to dietary habits [16].

Although previous studies have explored avian microbiomes, there is a lack of integrated analysis focusing on both oral and cloacal microbiota in nectarivorous parrots under controlled captive conditions. Existing literature has primarily emphasized gut microbiota, often overlooking the oral cavity, which plays a critical role in early microbial colonization and dietary interactions. Furthermore, the combined influence of age and anatomical site on microbial diversity in *T. haematodus* has not been systematically investigated. The absence of high-resolution sequencing approaches, such as full-length *16S rRNA* gene analysis, further limits accurate taxonomic and phylogenetic characterization. These gaps restrict the understanding of host–microbiome interactions, particularly in relation to dietary adaptation, health monitoring, and conservation strategies in captive breeding systems.

Therefore, this study aimed to identify, characterize, and compare the oral and cloacal microbiomes of adult and juvenile *T. haematodus* maintained under captive conditions using full-length 16S rRNA sequencing with Oxford Nanopore Technology (ONT). By evaluating microbial composition across different age groups and anatomical sites, this study seeks to elucidate patterns of microbial diversity, determine dominant taxa, and assess ecological associations between diet and microbiome structure. The findings are expected to provide a comprehensive understanding of microbiome dynamics in nectarivorous parrots and contribute to improving captive nutrition, health management, and conservation strategies.

## MATERIALS AND METHODS

### Ethical approval

All experimental procedures involving animals were reviewed and approved by the Animal Ethics Committee of the Indonesian Agency for Research and Innovation (BRIN), Bogor, Indonesia (Approval Number: 020/KE.02/SK/8/2022). The study was conducted in full compliance with national animal welfare regulations and internationally accepted ethical guidelines, including the ARRIVE guidelines 2.0 and the standards established by the World Organization for Animal Health (WOAH).

The study involved non-invasive sampling procedures using oral and cloacal swabs, which did not cause pain, injury, or long-term distress to the animals. Handling of *T. haematodus* was performed by trained personnel under the supervision of a licensed veterinarian to minimize stress and ensure animal welfare. No surgical procedures, anesthesia, or euthanasia were required during the study.

All birds were clinically healthy and sourced from a monitored captive population. Prior to inclusion, animals were assessed by a veterinarian to confirm the absence of disease or abnormal physiological conditions. Throughout the study period, birds were maintained under controlled environmental conditions and monitored daily for behavioral changes, physical abnormalities, and signs of distress. Any indication of compromised welfare would have resulted in immediate veterinary intervention; however, no such events occurred.

Housing, feeding, and environmental enrichment were provided in accordance with best practices for captive avian management. The study design adhered to the principles of the 3Rs (Replacement, Reduction, and Refinement), using the minimum number of animals required to achieve scientific objectives while ensuring high standards of care and minimizing animal use.

No endangered or protected individuals were harmed, and the study did not involve wild capture or invasive manipulation. All procedures were conducted in a manner that ensured the highest level of ethical responsibility, animal welfare, and scientific integrity.

### Study period and location

The study was conducted from July to October 2024 at a controlled research facility under the Indonesian Agency for Research and Innovation (BRIN), Bogor, Indonesia. The experimental and sampling activities were performed during a continuous 14-day observation period under standardized environmental conditions.

### Study design

**Figure 1:**
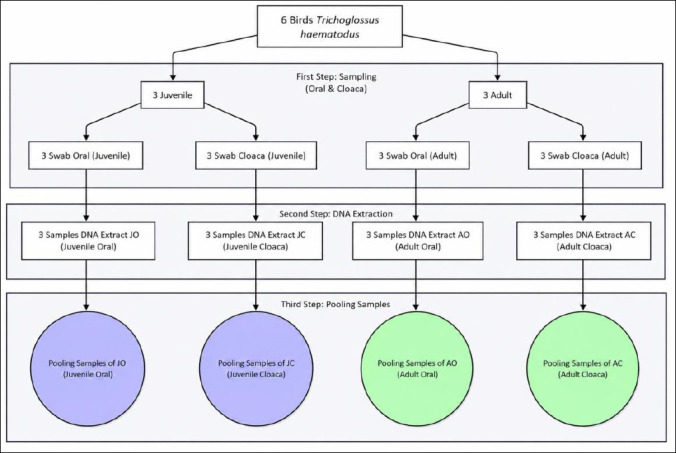
Schematic diagram of sampling, DNA extraction, and pooling samples

This study employed a controlled experimental design involving six clinically healthy *Trichoglossus haematodus*, consisting of three adults and three juveniles. Birds were maintained under identical housing, environmental, and dietary conditions. Samples were collected from two anatomical sites (oral and cloacal) and categorized into four groups: adult oral (AO), adult cloaca (AC), juvenile oral (JO), and juvenile cloaca (JC) (Figure 1). The study focused on comparative microbiome characterization using full-length 16S rRNA sequencing and descriptive bioinformatic analysis.

### Bird care and housing

A total of six *T. haematodus* (three adults and three juveniles) were housed individually in cages (70 cm × 43 cm × 52 cm) at a controlled temperature of 29.9 ± 1.1°C. During captivity, birds were provided with a diverse diet including fruits (apple, mango, papaya, banana, guava), seeds (fresh corn, sunflower seeds, cooked edamame), vegetables (bean sprouts, cucumber, long bean), and commercial baby biscuits soaked in brown sugar solution. Feeding was performed daily at 9:00 am, and water was provided *ad libitum*.

Feed intake for each bird was recorded daily over 14 days. The quantities of feed offered and remaining were weighed, and intake was calculated as the difference between these values. All birds were sourced from a monitored population and showed no clinical signs of disease. Additional diagnostic screening was deemed unnecessary by the attending veterinarian. Health monitoring included daily observation of morphology, behavior, and clinical signs of stress or disease. All animals remained under continuous veterinary supervision throughout the study.

### Sampling and handling

Six *T. haematodus* (three adults and three juveniles) were maintained under identical environmental and dietary conditions. Oral and cloacal swab samples were collected from each bird using sterile cotton swabs approximately 2 h after feeding. A total of 12 samples were obtained, including three JO, three JC, three AO, and three AC samples.

Each swab was placed into a sterile tube containing 10% glycerol as a cryoprotectant and stored at −80°C for long-term preservation until DNA extraction.

### Sex determination

Sex determination was performed using polymerase chain reaction (PCR) with universal primers 2550F (5’-GTTACTGATTCGTCTACGAGA-3’) and 2718R (5’-ATTGAAATGATCCAGTGCTTG-3’). These primers target the CHD gene, which exists in two forms: CHD-Z (Z chromosome) and CHD-W (W chromosome). Breast feather samples (plumae) were used for DNA extraction.

### DNA extraction

DNA extraction was performed individually for each sample before pooling. Extraction was carried out using the QIAamp Fast DNA Stool Mini Kit (Qiagen, Hilden, Germany) according to the manufacturer’s protocol with modifications, including treatment with lysozyme (25 mg/mL) (Calbiochem, Millipore, USA and Canada), RBC lysis buffer, and RNase (10 µL/mL) (Geneaid Biotech Ltd., Taipei, Taiwan).

This process yielded three JO, three JC, three AO, and three AC DNA extracts. DNA concentration and purity were measured using a nanophotometer (Implen, Munich, Germany). After quality assessment, equal amounts of DNA from individuals within each group were pooled, resulting in four composite samples: JO, JC, AO, and AC.

### Bioinformatic pipeline

Sequencing libraries were prepared following standard protocols. Sequencing was performed using ONT with the PromethION platform. Raw signal data were acquired using MinKNOW software version 24.02.16 in FAST5 format. Basecalling was conducted using Dorado version 7.3.11 [18], generating FASTQ files under a high-accuracy model (Table 1).

**Table 1:** Summary of raw and filtered sequencing data.

Group	n	Raw data (reads)	Mean read length (bp)	Filtered data (reads)	Mean read length (bp)
AO	3	102,700.0	1,589.7	93,712.0	1,610.0
JO	3	102,700.0	1,593.8	94,773.0	1,609.5
AC	3	105,846.0	1,576.9	94,803.0	1,610.9
JC	3	104,204.0	1,563.4	91,525.0	1,625.0

Quality control was performed using NanoPlot [19, 20] to evaluate read length and quality distribution. Reads were filtered using NanoFilt version 1.8.0 (https://github.com/wdecoster/nanoflt), with a minimum read length of 1000 bp and minimum quality score threshold. High-quality reads were taxonomically classified using Centrifuge against the SILVA 16S rRNA reference database with a confidence threshold of 0.7 [21].

Taxonomic abundance was visualized using Pavian, and radial community structures were generated using KronaTools. Downstream analyses included alpha diversity (Observed operational taxonomic unit (OTUs), abundance-based coverage estimator (ACE), Simpson, and Fisher) and beta diversity (Venn diagram and principal coordinate analysis (PCoA)) using R software version 4.2.3 (https://www.R- project.org/). No inferential statistical tests were performed; all analyses were descriptive.

### PCR product visualization

**Figure 2:**
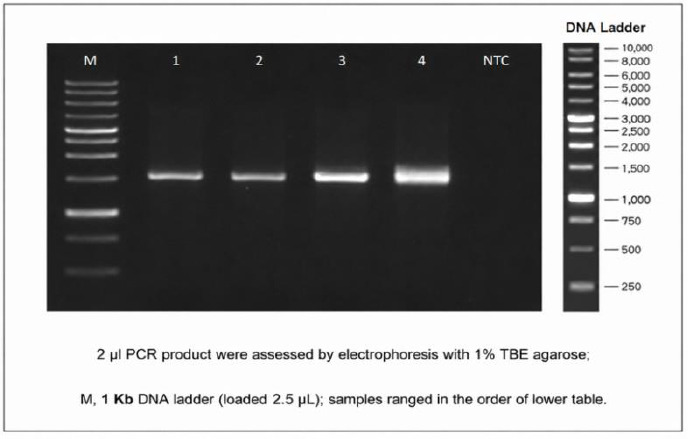
Polymerase chain reaction product (amplification of gDNA with primer 16S rRNA 27F–1492R).

PCR products were analyzed using gel electrophoresis on 1% TBE agarose. Migration patterns of samples (lanes 1–4) were compared with a 1 Kb DNA ladder (lane M) to determine fragment size. The non-template control confirmed the absence of contamination (Figure 2).

### Statistical analysis

This study used a bioinformatics-based metagenomic approach. Sequencing data were processed through ONT pipelines including MinKNOW, Dorado, NanoPlot, and NanoFilt version 1.8.0 (https://github.com/ wdecoster/nanoflt). Taxonomic classification was performed using Centrifuge with the SILVA 16S rRNA database, followed by downstream analysis using Pavian https://github.com/fbreitwieser/pavian), KronaTools (https:// github.com/marbl/Krona), and R software version 4.2.3 (https://www.R- project.org/).

Alpha diversity (Observed OTUs, ACE, Simpson, and Fisher) and beta diversity (Venn diagram and PCoA) were calculated using R Studio (version 4.2.3). No inferential statistical analysis was conducted, and all results are presented descriptively.

## RESULTS

### Biological characteristics of experimental birds

The biological data of the experimental subjects are presented in Table 2. The data include sex, age, body weight, and rearing period for both juvenile and adult groups.

**Table 2:** Biological data of juvenile and adult *Trichoglossus haematodus*.

Group	ID	Sex	Age	Weight	Rearing period
Juvenile*	J1	Male	2 months	115 g	2 months
	J2	Female	3 months	117 g	3 months
	J3	Male	2 months	113 g	2 months
Adult**	A1	Male	Adult	140 g	± 3 years
	A2	Female	Adult	130 g	± 3 years
	A3	Female	Adult	130 g	± 3 years

* Born in research facility,

** Born in commercial facility

### Feed intake patterns

The average daily feed intake data for juvenile and adult *T. haematodus* for all feed types are presented in Table 3. The results show variations in consumption depending on feed type and age group during the 14-day observation period.

**Table 3:** Daily feed intake of juvenile and adult *T. haematodus* (g/bird/day).

Feed	Juvenile (X ± sd)	Adult (X ± sd)
Apple	0.34 ± 0.16	2.78 ± 0.44
Mango	0.71 ± 0.32	1.87 ± 0.40
Papaya	2.04 ± 1.29	2.25 ± 0.36
Banana	1.11 ± 0.71	2.84 ± 0.76
Guava	1.78 ± 0.27	1.72 ± 0.33
Fresh corn	3.78 ± 0.89	5.95 ± 2.83
Sunflower seed	0	2.97 ± 0.51
Cooked edamame	1.18 ± 0.81	4.97 ± 0.63
Bean sprouts	0.14 ± 0.01	1.65 ± 0.40
Cucumber	2.05 ± 1.89	3.98 ± 1.86
Long bean	0	1.83 ± 0.57
Baby biscuit in nectar solution	47.12 ± 10.94	70.34 ± 7.56

### Nutritional composition of feed

The nutritional content of *T. haematodus* feed, including baby biscuit in nectar solution, is presented in Table 4. The table summarizes macronutrients, vitamins, and mineral contents per 100 g fresh weight.

**Table 4:** Nutritional composition of *T. haematodus* feed (per 100 g fresh weight).

Nutrient	Unit	Apple*	Mango*	Papaya*	Banana*	Guava*	Fresh corn*	Sunflower seed*	Cooked edamame*	Bean sprouts*	Cucumber*	Long bean*	Baby biscuit in nectar
**Macronutrients**													
Water	gram	86.00	83.46	89.80	75.30	84.25	76.05	1.20	72.77	90.40	95.23	87.85	74.28
Total fat	gram	0.20	0.38	0.14	0.26	0.95	1.38	50.00	5.20	0.19	0.19	0.44	0.57
Protein	gram	0.26	0.85	0.60	0.78	2.55	3.24	19.53	12.00	3.08	0.58	2.80	0.40
Total carbohydrate	gram	13.81	15.50	10.98	22.61	14.32	18.62	24.22	8.91	5.96	3.65	8.35	24.66
Calories	kcal	0.05	0.06	0.04	0.10	0.07	0.09	0.58	0.14	0.03	0.02	0.05	105.33
Dietary fibers	gram	2.40	1.58	0.80	1.74	5.40	2.00	10.94	5.20	1.83	0.58	4.00	–
Sugar	gram	10.39	13.94	9.80	15.82	8.92	6.28	2.73	2.18	4.13	1.73	1.88	–
Ash	gram	0.30	0.36	0.70	0.70	0.65	0.62	5.60	1.21	0.44	0.38	0.58	0.10
**Micronutrients - Vitamins**													
Folate	mcg	3.00	43.00	55.00	23.60	49.00	42.00	237.00	311.00	61.00	7.00	62.00	122.64
Niacin (Vit B3)	mg	0.09	0.67	0.34	0.66	1.08	1.77	7.04	0.96	0.75	0.10	0.41	0.00
Riboflavin (Vit B2)	mg	0.03	0.10	0.07	0.00	0.04	0.06	0.25	0.16	0.12	0.03	0.11	0.57
Thiamin (Vit B1)	mg	0.02	0.03	0.05	0.06	0.07	0.16	0.11	0.20	0.08	0.03	0.11	0.00
Vitamin A	IU	208.39	180.00	156.70	3.33	103.32	29.97	0.00	866.66	3.33	16.65	143.19	235.34
Vitamin B6	mg	0.04	0.12	0.15	0.21	0.11	0.09	0.80	0.10	0.09	0.04	0.02	0.49
Vitamin C	mg	4.60	36.42	108.00	12.26	228.30	6.83	1.41	6.10	13.17	2.88	18.80	4.12
Vitamin E	mg	0.18	0.90	5.30	0.20	0.73	0.07	26.10	0.68	0.10	0.04	0.00	6.09
Vitamin K	mcg	2.20	4.18	2.90	0.09	2.60	0.28	2.73	26.70	33.08	16.35	41.60	5.09
**Micronutrients - Minerals**													
Calcium, Ca	mg	6.00	10.91	24.00	5.00	18.00	2.00	70.00	63.00	13.00	16.00	50.00	201.27
Copper, Cu	mg	0.03	0.11	0.14	0.10	0.23	0.06	1.83	0.35	0.16	0.04	0.05	0.00
Iron, Fe	mg	0.12	0.16	0.66	0.00	0.26	0.52	3.80	2.27	0.91	0.29	0.47	6.09
Magnesium, Mg	mg	5.00	10.00	33.00	28.00	22.00	37.00	129.00	64.00	21.00	13.00	44.00	28.24
Manganese, Mn	mg	0.03	0.06	0.03	0.26	0.15	0.16	2.11	1.02	0.19	0.08	0.21	0.33
Phosphorus, P	mg	11.00	14.00	33.00	22.00	40.00	89.00	1155.00	169.00	54.00	24.00	59.00	78.63
Potassium, K	mg	107.00	168.00	257.00	326.00	417.00	270.00	850.00	436.00	149.00	147.00	240.00	0.00
Selenium, Se	mcg	0.00	0.60	1.50	0.00	0.60	0.60	79.30	0.80	0.60	0.31	1.50	0.00
Sodium, Na	mg	1.00	1.00	3.00	0.00	2.00	15.00	3.00	6.00	6.00	2.00	4.00	0.00
Zinc, Zn	mg	0.04	0.09	0.09	0.16	0.23	0.46	5.29	1.37	0.41	0.19	0.37	2.45

### Overall microbial diversity

The gut microbiota of *T. haematodus* was evaluated using cloacal and oral samples to assess age-related differences. A total of 1859 bacterial species were identified across all groups, indicating substantial microbial diversity influenced by both age and sampling site.

### Alpha diversity analysis

**Figure 3:**
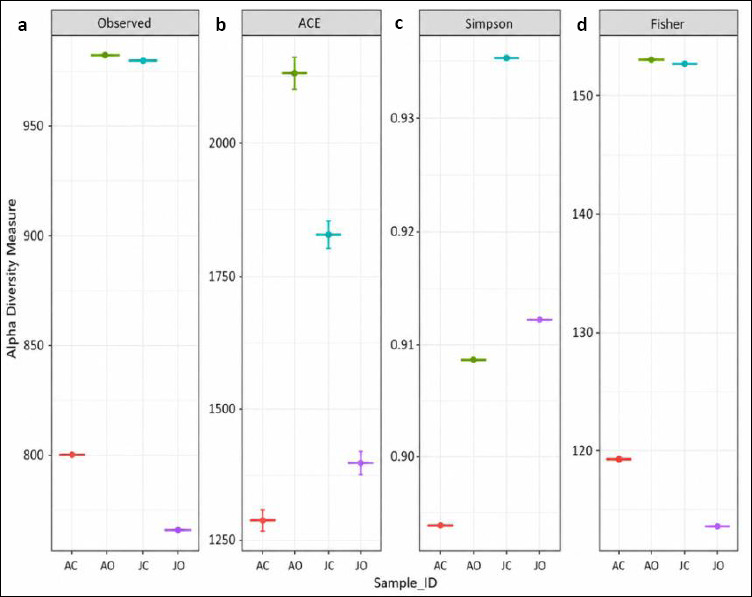
General information of alpha diversity analysis of bacterial gut microbiome of *Trichoglossus haematodus*: (a) Observed operational taxonomic units; (b) abundance-based coverage estimator; (c) Simpson index; (d) Fisher index. AC: Adult cloaca; AO: Adult oral; JC: Juvenile cloaca; JO: Juvenile oral.

Alpha diversity of the gut microbiome in *T. haematodus* was measured using observed OTUs, ACE, Simpson, and Fisher indices (Figure 3). The results demonstrate variations in bacterial diversity across the four sample groups.

Observed OTUs showed that JC and AO had similar values (~980) and the highest richness, whereas AC and JO exhibited lower values (~800). The ACE index indicated higher richness in JC (1829) and AO (2131), while AC and JO showed lower richness (1283 and 1358, respectively). The Simpson index demonstrated that JC had the highest diversity value, whereas JO and AO had similar values (0.91), and AC showed the lowest diversity. The Fisher index indicated higher diversity in AO (153.00) and JC (1522.70), with AC and JO showing lower values (119.35 and 113.21, respectively).

### Beta diversity analysis

**Figure 4:**
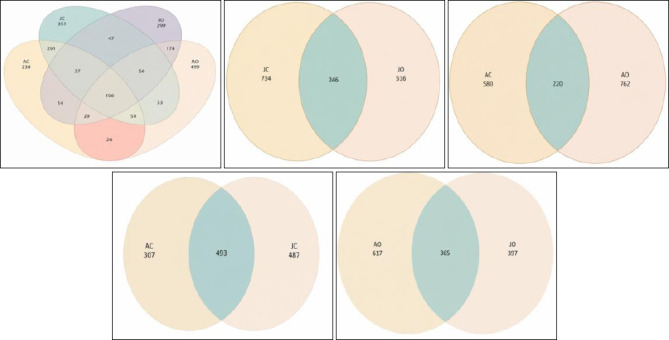
Venn diagram showing relationships between samples at the species level: (a) all samples; (b) JC vs JO; (c) AC vs AO; (d) AC vs JC; (e) AO vs JO. AC: Adult cloaca; AO: Adult oral; JC: Juvenile cloaca; JO: Juvenile oral.

Beta diversity of microbiota samples in *T. haematodus* is illustrated using Venn diagrams (Figure 4). A total of 106 OTUs were shared across all samples. Unique OTUs were observed as follows: AC (238), JC (351), JO (299), and AO (499).

JC and JO shared 246 core OTUs, whereas AC and AO shared 220 OTUs. JC exhibited higher unique OTUs than JO (734 vs 516), while AC had fewer unique OTUs than AO (580 vs 762). Furthermore, AC and JC shared 493 OTUs, and AO and JO shared 365 OTUs. AC had fewer unique OTUs than JC (307 vs 487), whereas AO had more unique OTUs than JO (617 vs 397).

### Microbial community composition

**Figure 5:**
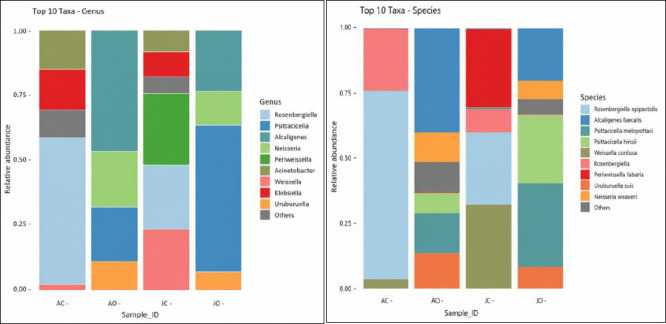
(a) Relative abundance of bacterial communities at the genus level (top 10 genera). (b) Relative abundance of bacterial communities at the species level (top 10 species).

The relative abundance of bacterial communities was analyzed at genus and species levels (Figures 5a and b). Microbial composition differed across AO, AC, JO, and JC groups.

At the genus level, *Rosenbergiella* was the dominant genus in AC (56.3%), followed by *Klebsiella* (15.1%) and *Acinetobacter* (15.6%). AO showed a more balanced profile, dominated by *Alcaligenes* (46.5%), followed by *Neisseria* (21.3%), *Psittacicella* (20.5%), and *Uruburuella* (10.8%).

JC exhibited a more complex microbial structure, dominated by *Periweissella* (27.3%), *Rosenbergiella* (24.8%), and *Weissella* (22.3%), with smaller contributions from *Klebsiella* (8.9%) and *Acinetobacter* (8.8%). In JO, *Psittacicella* (56.4%) was dominant, followed by *Alcaligenes* (23.8%), *Neisseria* (12.8%), and *Uruburuella* (6.3%).

At the species level, AC was dominated by *Rosenbergiella epipactidis* (73.0%), followed by *Rosenbergiella* spp. (23.1%) and *Weissella confusa* (3.2%). AO showed higher diversity, with Alcaligenes faecalis (40.3%) as dominant, followed by *Psittacicella meloposticti* (15.4%), *Uruburuella suis* (12.8%), *Neisseria weaveri* (11.5%), and *P. hinzii* (7.9%).

JC was dominated by *W. confusa* (31.7%), *Periweissella fabaria* (30.1%), and *R. epipactidis* (28.2%). JO showed higher complexity, with *P. meloposticti* (32.2%), *P. hinzii* (26.5%), *A. faecalis* (20.4%), and *U. suis* (7.5%).

### Heatmap visualization of microbial abundance

**Figure 6:**
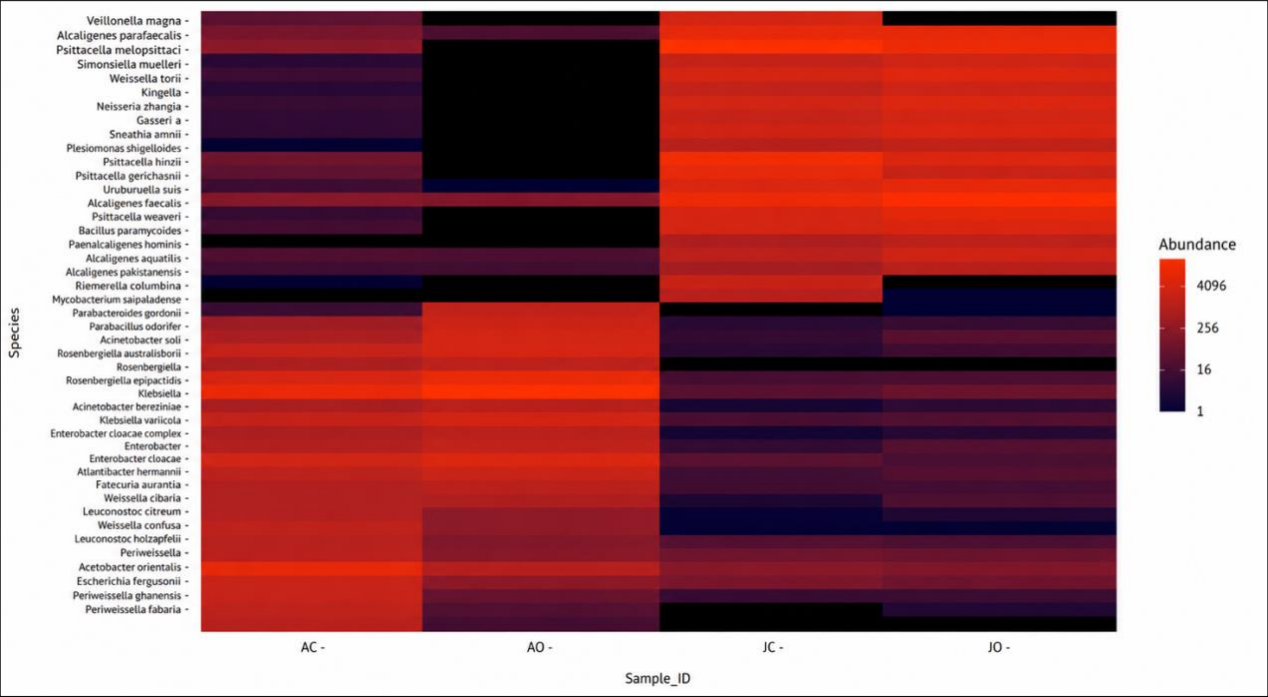
Heatmap of relative abundance of *T. haematodus* gut microbiome at species level.

The top 50 OTUs were visualized using a heatmap (Figure 6). Color intensity reflects microbial abundance, with lighter colors indicating higher abundance. Distinct microbial patterns were observed across age groups and sampling sites. *R. epipactidis* was most abundant in cloacal samples, particularly AC, whereas *P. meloposticti* dominated oral samples. *W. confusa* and *P. fabaria* were consistently observed across all groups.

### Targeted microbial populations

The specific pathogenic bacteria taxa identified across all samples, along with their associated references are summarized in Table 5 [22, 24–30]. This table provides a detailed overview of these potential pathogens, highlighting their relevance to the health monitoring of captive birds.

**Table 5:** Population of targeted gut microbes

Genus	Adult cloaca	Adult oral	Juvenile cloaca	Juvenile oral	Reference
*Salmonella*	59	1	226	1	[22–24]
*Escherichia*	19	0	1692	0	[22]
*Clostridium*	1	7	1042	3	[25]
*Pseudomonas*	27	0	29	0	[25]
*Staphylococcus*	8	282	12	40	[22, 24, 26]
*Klebsiella*	10539	33	5625	41	[27]
*Campylobacter*	1	0	0	0	[28]
*Lactococcus*	55	9	519	87	[22, 29]
*Enterococcus*	23	0	9	2	[22, 30]

### PCoA analysis

**Figure 7:**
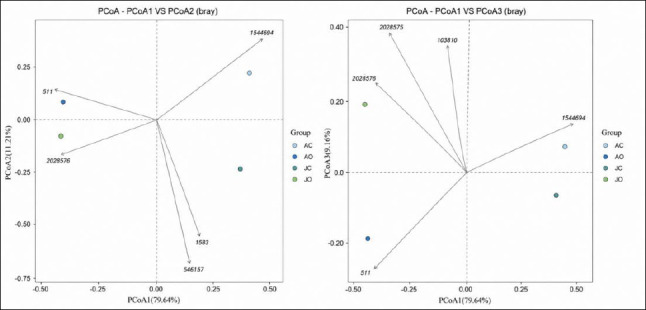
PCoA plots based on Bray–Curtis dissimilarity.

PCoA analysis demonstrated distinct clustering patterns among sample groups (Figure 7). The first axis explained 79.64% of variance, while the second and third axes explained 11.21% and 9.16%, respectively. These results indicate clear separation among microbial communities, primarily driven by sampling site and age.

## DISCUSSION

### Age- and site-related variation in microbial diversity

Based on the present results, the oral microbiota of adult *T. haematodus* (AO) exhibited higher species richness, as indicated by the ACE index, but showed lower evenness. This pattern may reflect the dominance of a few bacterial taxa, resulting in a less balanced community structure. This finding is consistent with observations in other vertebrates, in which older individuals often maintain a more stable but less diverse microbiota, possibly due to established ecological niches and age-associated phenotypes, including oxidative stress and proinflammatory responses [31].

Conversely, the oral microbiota of juvenile *T. haematodus* (JO) showed a more balanced microbial distribution, characterized by greater taxonomic diversity. Younger animals tend to harbor more varied microbiota and often show higher alpha diversity indices than adults [32, 33]. Studies on captive zebra finches and Bengalese finches have also indicated that juveniles have higher alpha diversity than adults. Bacteria in juveniles may represent early microbial colonizers adapting to the host because the immune system is not yet fully developed [34]. The presence of diverse bacteria in juvenile *T. haematodus* may reflect developmental processes in which a rich microbial community provides essential metabolic functions and improves resilience against pathogens [35].

The cloacal microbiota of adult (AC) and juvenile (JC) *T. haematodus* showed similar community structures, characterized by high evenness and a more balanced distribution of taxa. Compared with the oral microbiota, the cloacal microbiota appeared more stable across age groups. Microbiota diversity in the cloaca of parrots was higher than that in the oral cavity [36]. The higher diversity observed in the cloacal microbiota may be associated with feed breakdown into nutrients, which can influence the gut environment and provide substrates for bacterial growth.

### Microbial stability and developmental effects in juveniles

The present results also indicated that the gut microbiome of juvenile birds remains strongly influenced by environmental factors and feeding behavior. The high alpha diversity observed in juvenile cloacal samples of *T. haematodus* suggests that juvenile birds have not yet achieved microbial stability in the digestive tract. Lower alpha diversity in adult cloacal samples indicates that microbial stability develops as birds mature.

Gastrointestinal tracts of juvenile birds are initially colonized by numerous transient bacterial species, which gradually develop into a more stable adult-like community [36]. In addition to bacteria, other studies have shown that juvenile birds harbor more transient viral species, probably acquired early in life, whereas adults exhibit a more stable viral community. These changes in microbial diversity reflect the influence of age on both bacterial and viral communities within avian hosts [37]. Meanwhile, the high alpha diversity observed in the oral microbiome of adult birds may indicate more varied feeding behavior in adults than in juveniles [38].

### Ecological relevance of *Rosenbergiella* in nectar-feeding birds

*T. haematodus* primarily consumes nectar and pollen from flowers. Based on Figure 5b, *Rosenbergiella* species, such as *R. epipactidis*, are Gram-negative, rod-shaped, and facultatively anaerobic bacteria. They have specialized growth characteristics, including temperature and salinity tolerance, that allow them to thrive in nectar habitats [39]. The microbial community of *R. epipactidis* and related species has evolved to survive in the unique chemical environment of floral nectar. Several factors affect their growth, including sugar concentration and nitrogen availability in nectar [40]. Although *R. epipactidis* is most commonly associated with floral nectar, bacteria perform critical functions in multiple ecosystems, including soil and aquatic environments. Their metabolic flexibility allows them to participate in biogeochemical cycles and adapt to diverse environments [41].

*Rosenbergiella* belongs to the family *Enterobacteriaceae*, which is commonly isolated from floral nectar. The high abundance of *Rosenbergiella* in cloacal samples of lorikeets may be associated with their role as pollinators or nectarivores [25]. In addition, the abundance of Enterobacteriaceae in lorikeets may indicate susceptibility to enteritis. Captive lorikeets are commonly fed commercial nectar powder diets that contain trypsin inhibitors. High levels of trypsin inhibitors in lorikeet diets may play a major role in susceptibility to enteritis [25]. Fortunately, in the present study, some taxa predictive of enteritis susceptibility, including *Rhodococcus fascians*, *Kocuria* spp., *Pseudomonas umsongensis*, and *Aeromonas* spp., were present at low abundance in cloacal samples of lorikeets.

*Rosenbergiella* is commonly detected in nectarivorous birds, such as hummingbirds [42]. However, it has rarely been identified as a specific bacterium in parrots. One study detected *Rosenbergiella* in a captive Alexandrine parakeet (*Palaeornis eupatria*), but did not specifically discuss *Rosenbergiella* [43].

### Influence of habitat, diet, and anatomical site on microbiome composition

Microbiome diversity is strongly influenced by environmental factors, including habitat and lifestyle. Birds with broader habitat ranges and more diverse diets typically have more diverse microbiomes [44]. The diversity of microbiota in birds differs substantially between the digestive tract and oral cavity because of differences in pH, enzymes, and organic substrates available in each habitat [45]. Dominant bacteria in the oral cavity of vertebrates often belong to Firmicutes and Bacteroidetes, whereas Proteobacteria are usually more common in the cloaca or digestive tract. These phyla are frequently found in avian gut ecosystems and play important roles in digestion and immune function. Differences in the dominance of these genera or species can be influenced by diet, lifestyle, and environmental conditions [46, 47].

### Potential probiotic relevance of *Weissella* and other commensal bacteria

The presence of *Weissella* in juvenile cloacal samples highlights the importance of microbial diversity in maintaining gut health and preventing pathogen colonization. Most *Weissella* species meet the prerequisites required to be categorized as probiotics [48]. In parrots, high abundance of *Leuconostoc* and *Weissella* and low levels of *Clostridium* and *Escherichia*/*Shigella* have been correlated with weight gain. Moreover, anorexia has been associated with higher abundance of *Kocuria* and *Streptococcus* [49].

The gut microbiome is crucial for avian health because it influences disease resistance and overall well-being. In chickens, modulation of the gut microbiota has been shown to alleviate infections, highlighting the potential role of microbiome management in disease control [22]. Microbiomes also contribute to ecological interactions, including pathogen transmission. Migratory birds can act as reservoirs for pathogens, posing risks to other species and humans [50, 51]. Similar to mammalian systems, the gut microbiota supports immune system function and nutrient absorption. It contributes to general physiological health and assists in the processing of pollutants [52]. The microbiome also protects against pathogens, thereby supporting disease resistance. However, wild birds can harbor antibiotic-resistant bacteria, creating a risk of disease transmission and resistance spread [53].

### Pathogenic bacterial taxa and implications for captive health monitoring

The gut microbiome of wild birds is highly diverse and dynamic, playing a crucial role in digestion, immune function, and overall health. Multiple factors, including diet, age, environmental conditions, antibiotic use, and pathogen exposure, can shape the composition of avian gut microbiota [54]. However, among the many microorganisms present in the intestine, some may act as enteropathogens and potentially cause gastrointestinal infections and other health issues [55]. These pathogenic bacteria include *Salmonella*, *Escherichia*, *Clostridium*, *Pseudomonas*, *Staphylococcus*, *Klebsiella*, and *Campylobacter* (Table 5).

Among these taxa, *Klebsiella* was the most abundant in the cloaca of both adult and juvenile birds. Wild birds are known carriers of *Klebsiella*, and microbial studies have confirmed its presence in their intestines. The prevalence of these bacteria in wild birds is influenced by diet, environmental exposure, and interactions with contaminated water, soil, or human-associated waste. In addition, *Klebsiella* forms part of the intestinal microbiota of humans and animals and has been found in several domestic and wild animal species, including mammals, fish, birds, mollusks, insects, and earthworms [27]. In the present study, the adult cloacal sample showed high prevalence of *Klebsiella*. In young birds, bacterial flora is still developing, and microbial diversity tends to increase as certain avian species mature [56].

Meanwhile, the prevalence of *Salmonella*, *Escherichia*, and *Clostridium* was higher in the cloaca of juvenile birds than in adults. The higher prevalence of *Clostridium*, *Escherichia*, and *Salmonella* in juvenile cloacal samples may be linked to environmental conditions, developmental processes, and ecological interactions. Juvenile birds have less developed immune systems than adults, making them more susceptible to pathogen colonization [57]. This immune immaturity can result in higher bacterial loads in the cloaca because juvenile birds are less capable of clearing infections.

Identification of *Salmonella*, *Escherichia*, and *Klebsiella* through complete 16S rRNA sequencing provides an important basis for health monitoring in captive populations. Although these taxonomic profiles detect bacterial DNA, they do not distinguish between commensal strains and strains carrying active virulence factors. However, the high abundance of *Klebsiella* in adult cloacal samples and the presence of *Salmonella* in juveniles are important ecological indicators of potential zoonotic risk and susceptibility to enteritis. This finding highlights the need for biosecurity and hygiene protocols for breeders in captive breeding facilities.

Furthermore, dietary changes that cause loss of host flexibility and metabolic shifts are frequently observed in captive breeding programs [58]. Captive birds can also spread antibiotic-resistant bacteria and zoonotic pathogens; therefore, mapping the gut microbiome of birds intended for reintroduction has become a useful tool for wildlife conservation and the One Health concept [59].

### Antibiotic resistance and One Health relevance

Several recent studies have demonstrated that captivity and environmental transition can substantially influence the prevalence and distribution of antibiotic resistance in wildlife. One study identified that rehabilitation and environmental changes in migratory birds were associated with increased antibiotic resistance, including the emergence of multidrug resistance in *E. coli*. Similarly, captive musk deer (*Moschus berezovskii*) showed higher diversity and abundance of antibiotic resistance genes than wild deer based on metagenomic and culture-based methods [60, 61].

Young birds may have different diets and foraging behaviors than adults, which can increase their exposure to contaminated feed sources. The cloacal microbiota of juvenile birds is often more diverse and less stable than that of adults. This diversity may include a higher prevalence of pathogenic bacteria, such as *Escherichia* and *Salmonella*, which may not persist in the microbiota of older birds because of the establishment of a more stable microbial community [23, 57].

*Staphylococcus* species are frequently present on the skin, feathers, and mucosal surfaces of wild birds, including the beak and oral cavity. These bacteria are natural components of the avian microbiota and can be isolated from various body regions. In a previous study, *Staphylococcus aureus* was recovered from the oral cavity of racing pigeons showing lesions characteristic of pigeon pox virus infection [26].

Commensal bacteria are integral components of the gut microbiome in wild birds and contribute to physiological functions such as digestion and immune modulation. Among these, *Enterococcus* species are frequently detected. A study analyzing fecal samples from wild birds reported a high occurrence of Enterococcus species, with *Enterococcus faecalis* being the most predominant, followed by *E. hirae* and *E. faecium* [30]. *Lactococcus* species have been shown to modulate the gut microbiota and produce beneficial effects for the host. In rats, Lactococcus petauri substantially modified the gut microbial community by enhancing short-chain fatty acid producers, which increased the production of total short-chain fatty acids, acetic acid, and propionic acid [29].

### PCoA-based interpretation of community structure

The separation along PCoA1 suggests that this axis represents a key ecological gradient influencing microbial community composition. Clustering of samples within the same group indicates that community structures are more similar within groups than between groups, suggesting that specific environmental or biological factors shape microbial composition. PCoA results in migratory birds indicated that the microbial structure of migratory birds was more heterogeneous than that of environmental samples [62]. Overall, this pattern suggests that the main factor distinguishing the groups plays a significant role in shaping community structure. Samples within the same group tended to cluster together because of shared characteristics, whereas clear separation between groups reflected distinct compositional differences.

There was clear separation of sample points, suggesting that the community composition of AC, AO, JC, and JO differed distinctly. Clustering of points within the same group indicated compositional similarity, whereas larger distances between groups suggested substantial differences. Separation along PCoA1 indicates that this axis represents the most influential factor differentiating the groups, which may include environmental conditions, species interactions, or external influences. Gut microbes can influence phenotypic traits of animals, whereas metabolite concentrations in the gut regulate microbial functions [43, 63]. In this study, dietary changes through pellet feeding and age directly affected the intestinal microbial community and metabolites in Alexandrine parrots [43].

## CONCLUSION

This study provides the first comparative characterization of oral and cloacal microbiomes in captive adult and juvenile *T. haematodus* using full-length 16S rRNA sequencing. A total of 1859 bacterial species were identified across the four groups, demonstrating that microbial diversity and community composition differed according to age and anatomical site. Cloacal samples showed greater stability between age groups, whereas oral samples reflected stronger age-related variation. AO samples showed higher richness, while juvenile cloacal samples exhibited the highest diversity. At the genus level, *Rosenbergiella* dominated cloacal samples, particularly in adults, whereas *Alcaligenes* and *Psittacicella* were dominant in adult and JO samples, respectively. At the species level, *R. epipactidis*, *A. faecalis*, *P. meloposticti*, *W. confusa*, and *P. fabaria* were important contributors to group-specific microbial profiles.

The dominance of nectar-associated bacteria, especially *Rosenbergiella*, supports an ecological link between the nectar-based diet of *T. haematodus* and its cloacal microbiome. The detection of potentially pathogenic genera, including *Klebsiella*, *Salmonella*, *Escherichia*, *Clostridium*, *Pseudomonas*, *Staphylococcus*, and *Campylobacter*, also highlights the importance of routine microbiome-based health monitoring in captive breeding facilities. These findings have practical implications for improving captive nutrition, strengthening hygiene and biosecurity protocols, and supporting conservation programs for nectarivorous parrots.

A key strength of this study is the use of full-length 16S rRNA sequencing, which improved taxonomic resolution and enabled detailed comparison of microbial communities across age groups and anatomical sites. The controlled housing and standardized feeding conditions also reduced environmental variation and strengthened interpretation of age- and site-associated microbial patterns.

However, this study was limited by the small number of birds, pooled sample design, and descriptive analytical approach without inferential statistical testing. In addition, 16S rRNA sequencing cannot differentiate viable from non-viable bacteria or distinguish commensal strains from pathogenic strains carrying virulence or antimicrobial resistance genes.

Future studies should include larger sample sizes, individual-level sequencing, longitudinal sampling, functional metagenomics, antimicrobial resistance profiling, and culture-based validation. Comparative studies between wild and captive *T. haematodus* populations would also help clarify how captivity, diet, and environmental exposure shape microbiome development.

Overall, this study establishes baseline microbiome data for captive *T. haematodus* and demonstrates that age, anatomical site, and nectar-associated ecology are key factors shaping microbial communities. These findings provide a useful foundation for microbiome-informed health management, nutrition planning, and conservation strategies in captive nectarivorous parrots.

## DATA AVAILABILITY

All data generated or analyzed during this study are included in this published manuscript. Additional information, clarifications, or supplementary data can be obtained from the corresponding author upon reasonable request.

## AUTHORS’ CONTRIBUTIONS

RR, SNP, SP, AF, RTP, WH, and LS: Study design. RR, KAS, APS, SM, and WW: Sample collection. RR, KAS, SP, APS, and SM: Laboratory analysis. SS, AF, and RRI: Methodology development. KAS, SS, and LS: Data validation. SNP, RRI, RTP, WH, and LS: Supervision. All authors contributed to the writing of the manuscript and approved the final version.

## References

[1] Prijono SN, Rachmatika R (2020). Effect of sweetness level and amino acid composition of palm sugar on feed intake of *Trichoglossus haematodus* in captivity. Biosaintifika.

[2] Frankel TL, Avram D (2001). Protein requirements of rainbow lorikeets, *Trichoglossus haematodus*. Aust J Zool.

[3] Davis A, Major RE, Taylor CE (2015). The association between nectar availability and nectarivore density in urban and natural environments. Urban Ecosyst.

[4] Garcia-Mazcorro JF, Alanis-Lopez C, Marroquin-Cardona AG, Kawas JR (2021). Composition and potential function of fecal bacterial microbiota from six bird species. Birds.

[5] Haberecht S, Bajagai YS, Moore RJ, Van TTH, Stanley D (2020). Poultry feeds carry diverse microbial communities that influence chicken intestinal microbiota colonization and maturation. AMB Express.

[6] Zou A, Nadeau K, Xiong X, Wang PW, Copeland JK, Lee JY (2022). Systematic profiling of the chicken gut microbiome reveals dietary supplementation with antibiotics alters expression of multiple microbial pathways with minimal impact on community structure. Microbiome.

[7] Schweizer M, Güntert M, Seehausen O, Leuenberger C, Hertwig ST (2014). Parallel adaptations to nectarivory in parrots, key innovations and the diversification of the Loriinae. Ecol Evol.

[8] McWhorter TJ, Rader JA, Schondube JE, Nicolson SW, Pinshow B, Fleming PA (2021). Sucrose digestion capacity in birds shows convergent coevolution with nectar composition across continents. iScience.

[9] Napier KR, McWhorter TJ, Nicolson SW, Fleming PA (2013). Sugar preferences of avian nectarivores are correlated with intestinal sucrase activity. Physiol Biochem Zool.

[10] Nicolson SW, Fleming PA (2014). Drinking problems on a 'simple'diet: physiological convergence in nectar-feeding birds. J Exp Biol.

[11] Diaz Carrasco JM, Casanova NA, Fernández Miyakawa ME (2019). Microbiota, gut health and chicken productivity: What is the connection?. Microorganisms.

[12] Bajagai YS, Van TTH, Joat N, Chousalkar K, Moore RJ, Stanley D (2024). Layer chicken microbiota: A comprehensive analysis of spatial and temporal dynamics across all major gut sections. J Anim Sci Biotechnol.

[13] Aghili S, Rahimi H, Hakim LK, Karami S, Soufdoost RS, Oskouei AB (2024). Interactions between oral microbiota and cancers in the aging community: A narrative review. Cancer Control.

[14] Xiao J, Fiscella KA, Gill SR (2020). Oral microbiome: Possible harbinger for children's health. Int J Oral Sci.

[15] Lee YH, Chung SW, Auh QS, Hong SJ, Lee YA, Jung J (2021). Progress in oral microbiome related to oral and systemic diseases: An update. Diagnostics.

[16] Catassi G, Aloi M, Giorgio V, Gasbarrini A, Cammarota G, Ianiro G (2024). The role of diet and nutritional interventions for the infant gut microbiome. Nutrients.

[17] Bahram M, Anslan S, Hildebrand F, Bork P, Tedersoo L (2019). Newly designed 16S rRNA metabarcoding primers amplify diverse and novel archaeal taxa from the environment. Environ Microbiol Rep.

[18] Wick RR, Judd LM, Holt KE Performance of neural network basecalling tools for Oxford Nanopore sequencing.

[19] De Coster W, D'Hert S, Schultz DT, Cruts M, Van Broeckhoven C (2018). NanoPack: Visualizing and processing long-read sequencing data. Bioinformatics.

[20] Nygaard AB, Tunsjø HS, Meisal R, Charnock C (2020). A preliminary study on the potential of Nanopore MinION and Illumina MiSeq 16S rRNA gene sequencing to characterize building-dust microbiomes. Sci Rep.

[21] Kim D, Song L, Breitwieser FP, Salzberg SL (2016). Centrifuge: Rapid and sensitive classification of metagenomic sequences. Genome Res.

[22] Zhu J, Ding J, Yang K, Zhou H, Yang W, Qin C (2024). Microbiome and microbial pure culture study reveal commensal microorganisms alleviate Salmonella enterica serovar pullorum infection in chickens. Microorganisms.

[23] He Y, Zhang M, Dai C, Yu L (2023). Comparison of the gut microbial communities of domestic and wild mallards (Anas platyrhynchos) based on high-throughput sequencing technology. Animals.

[24] Cockerham S, Lee B, Orben RA, Suryan RM, Torres LG, Warzybok P (2019). Microbial ecology of the Western Gull (Larus occidentalis). Microb Ecol.

[25] Minich D, Madden C, Navarro MA, Glowacki L, French-Kim K, Chan W (2022). Gut microbiota and age shape susceptibility to clostridial enteritis in lorikeets under human care. Anim Microbiome.

[26] Chrobak-Chmiel D, Kwiecień E, Golke A, Dolka B, Adamczyk K, Biegańska MJ (2021). Pigeons as carriers of clinically relevant multidrug-resistant pathogens—A clinical case report and literature review. Front Vet Sci.

[27] Wyres KL, Holt KE (2016). Klebsiella pneumoniae population genomics and antimicrobial-resistant clones. Trends Microbiol.

[28] A One Health Perspective of Pet Birds Bacterial Zoonosis and Prevention (2024). Pak Vet J.

[29] Fang S, Qin T, Yu T, Zhang G (2022). Improvement of the gut microbiota in vivo by a short-chain fatty acids-producing strain Lactococcus garvieae CF11. Processes.

[30] Freitas ADAR, Faria AR, Mendes LT, Merquior VLC, Neves DM, Pires JR (2024). The gut microbiota of wild birds undergoing rehabilitation as a reservoir of multidrug-resistant enterococci in a metropolitan area in Brazil. Braz J Microbiol.

[31] Koh YC, Kuo LH, Tung YC, Weerawatanakorn M, Pan MH (2023). Identification of indicative gut microbial guilds in a natural aging mouse model. ACS Omega.

[32] De La Cuesta-Zuluaga J, Kelley ST, Chen Y, Escobar JS, Mueller NT, Ley RE (2019). Age- and sex-dependent patterns of gut microbial diversity in human adults. mSystems.

[33] Zhou L, Huo X, Liu B, Wu H, Feng J (2020). Comparative analysis of the gut microbial communities of the Eurasian kestrel (Falco tinnunculus) at different developmental stages. Front Microbiol.

[34] Maraci Ö, Antonatou-Papaioannou A, Jünemann S, Engel K, Castillo-Gutiérrez O, Busche T (2022). Timing matters: Age-dependent impacts of the social environment and host selection on the avian gut microbiota. Microbiome.

[35] JG, HY, TL, JJG, JRC, RW (2008). Effects of zinc bacitracin, bird age and access to range on bacterial microbiota in the ileum and caeca of broiler chickens. J Appl Microbiol.

[36] Van Dongen WF, White J, Brandl HB, Moodley Y, Merkling T, Leclaire S (2013). Age-related differences in the cloacal microbiota of a wild bird species. BMC Ecol.

[37] Wille M, Shi M, Hurt AC, Klaassen M, Holmes EC (2021). RNA virome abundance and diversity is associated with host age in a bird species. Virology.

[38] Yi T, Sun YH, Liang W (2020). Nestling discrimination and feeding habits during brooding of chestnut thrushes. Avian Res.

[39] Halpern M, Fridman S, Atamna-Ismaeel N, Izhaki I (2013). *Rosenbergiella* nectarea gen. nov., sp. nov., in the family Enterobacteriaceae, isolated from floral nectar. Int J Syst Evol Microbiol.

[40] Morales-Poole JR, De Vega C, Tsuji K, Jacquemyn H, Junker RR, Herrera CM (2023). Sugar concentration, nitrogen availability, and phylogenetic factors determine the ability of *Acinetobacter* spp. and *Rosenbergiella* spp. to grow in floral nectar. Microb Ecol.

[41] Semenov M, Li H, Luo Y, Deng Y, Kuzyakov Y (2023). Editorial: Microbial regulation of soil carbon cycling in terrestrial ecosystems. Front Microbiol.

[42] Lee C, Tell LA, Hilfer T, Vannette RL (2019). Microbial communities in hummingbird feeders are distinct from floral nectar and influenced by bird visitation. Proc R Soc B.

[43] Feng X, Zhu R, Luo C, Zhan T, Feng Y, Zhu Y (2024). Alterations in captive Alexandrine parakeet gut microbiome and metabolome in response to dietary change. Comp Biochem Physiol D Genomics Proteomics.

[44] Callaghan CT, Jurburg SD Bird life history traits influence the diversity of their associated microbiomes.

[45] Videvall E, Song SJ, Bensch HM, Strandh M, Engelbrecht A, Serfontein N (2020). Early-life gut dysbiosis linked to juvenile mortality in ostriches. Microbiome.

[46] Bukhari SM, Andleeb S, Alghamdi HA, Rehman KU, Javid A, Ali W (2024). Exploration of gut microbial diversity of pheasants through pyrosequencing of 16S rRNA gene. J Exp Zool A Ecol Integr Physiol.

[47] Sun M, Halimubieke N, Fang B, Valdebenito JO, Xu X, Sheppard SK (2024). Gut microbiome in two high-altitude bird populations showed heterogeneity in sex and life stage. FEMS Microbes.

[48] Ahmed S, Singh S, Singh V, Roberts KD, Zaidi A, Rodriguez-Palacios A (2022). The Weissella genus: Clinically treatable bacteria with antimicrobial and probiotic effects. Microorganisms.

[49] Černá K (2023). Interspecific and intraspecific variation in gastrointestinal microbiota composition of parrots and its association with incidence of selected disorders.

[50] Liu G, Xu N, Yu C (2024). Comparative analysis of the microbiome of sympatric wintering bean geese, domestic ducks, humans, and soil at Shengjin Lake of China reveals potential public risk to human health. Avian Res.

[51] Liu J, Li X, Song W, Zeng X, Li H, Yang L (2024). The multi-kingdom microbiome of wintering migratory birds in Poyang Lake, China. Viruses.

[52] Kohl KD (2012). Diversity and function of the avian gut microbiota. J Comp Physiol B.

[53] Rasmussen JA, Chua PYS (2023). Genome-resolving metagenomics reveals wild western capercaillies (Tetrao urogallus) as avian hosts for antibiotic-resistance bacteria. Microbiol Res.

[54] Fathima S, Shanmugasundaram R, Adams D, Selvaraj RK (2022). Gastrointestinal microbiota and their manipulation for improved growth and performance in chickens. Foods.

[55] Aruwa CE, Pillay C, Nyaga MM, Sabiu S (2021). Poultry gut health –microbiome functions, environmental impacts, microbiome engineering and advancements in characterization technologies. J Anim Sci Biotechnol.

[56] Wielen PWJJ, Keuzenkamp DA, Lipman LJA, Knapen F, Biesterveld S (2002). Spatial and temporal variation of intestinal bacterial communities in broiler chickens. Microb Ecol.

[57] Xu Q, Zhao W, Li Y, Zou X, Dong X (2022). Intestinal immune development is accompanied by temporal deviation in microbiota composition of newly hatched pigeon squabs. Microbiol Spectr.

[58] Wang W, Wang Y, Chen Q, Ding H (2023). Effects of diet shift on the gut microbiota of the critically endangered Siberian crane. Avian Res.

[59] Tufail A, Bo T, Zhao N, Willows-Munro S, Khan BN, Duan J (2025). Exploring microbiome shifts across taxonomic and ecological groups of birds at a key stopover site in Punjab, Pakistan. Curr Res Microb Sci.

[60] Song H, Yi S, Kim WH, Guk JH, Ha M, Kwak I (2022). Environmental perturbations during rehabilitation of migratory birds induce gut microbiome alteration and antibiotic resistance acquisition. Microbiol Spectr.

[61] Xi J, Tao H, Zhang Z, Lian B, Sun W, Zhang Y (2026). Captive breeding of specialty animals represents a critical reservoir for antibiotic resistance gene spread. ISME J.

[62] Cao J, Hu Y, Liu F, Wang Y, Bi Y, Lv N (2020). Metagenomic analysis reveals microbiome and resistome in migratory birds. Microbiome.

[63] Henderson G, Cox F, Ganesh S, Jonker A, Young W, Global Rumen Census Collaborators (2015). Rumen microbial community composition varies with diet and host. Sci Rep.

